# Lead contamination in Australian game meat

**DOI:** 10.1007/s11356-023-25949-y

**Published:** 2023-02-17

**Authors:** Jordan O. Hampton, Deborah J. Pain, Eric Buenz, Simon M. Firestone, Jon M. Arnemo

**Affiliations:** 1 Faculty of Science, University of Melbourne, Parkville, Victoria 3052 Australia; 2 Harry Butler Institute, Murdoch University, Murdoch, Western Australia 6150 Australia; 3 Department of Zoology, University of Cambridge, Cambridge, CB2 3QZ UK; 4 Nelson Marlborough Institute of Technology, Nelson, 7010 New Zealand; 5 Department of Forestry and Wildlife Management, Faculty of Applied Ecology and Agricultural Sciences, Inland Norway University of Applied Sciences, Koppang, Norway; 6 Department of Wildlife, Fish and Environmental Studies, Swedish University of Agricultural Sciences, Umeå, Sweden

**Keywords:** Ammunition, Australia, Dietary exposure, Food safety, Game meat, Heavy metals, Lead, Public health, Wildlife

## Abstract

Lead-based ammunition (gunshot and bullets) frequently leaves small lead fragments embedded in the meat of wild-shot game animals. Australia produces several commercial game meat products from wild animals harvested with lead-based ammunition and has a growing population of recreational hunters. However, no studies have previously investigated the frequency of lead fragments or lead concentrations in Australian game meat. We examined 133 Australian minced game meat items of four types for evidence of lead contamination. Samples were meat from kangaroos (*Macropus* and *Osphranter* spp.; *n*=36) and Bennett’s wallabies (*Notamacropus rufogriseus*; *n*=28) sold for human consumption, and deer (‘venison’; multiple spp.; *n*=32) and stubble quail (*Coturnix pectoralis*; *n*=37) harvested for private consumption by recreational hunters. All packages were studied by digital radiography to detect the presence of radio-dense fragments, assumed to be lead fragments from ammunition. Visible fragments were absent in commercially available kangaroo products, but were present in 4%, 28% and 35% of wallaby, venison and quail, respectively. Mean meat lead concentrations (mg/kg wet weight) were 0.01 ± 0.01 for kangaroo, 0.02 ± 0.01 for wallaby, 0.12 ± 0.07 for venison, and 1.76 ± 3.76 for quail. The Australian food standards threshold for livestock meat (0.1 mg/kg w.w.) was not exceeded by any kangaroo or wallaby products but was exceeded by 53% and 86% of venison and quail, respectively. Radiography only detected 35% of samples that were above the food safety threshold. While average lead concentrations in commercially available macropod (kangaroo and wallaby) meat were low, those in recreationally harvested game meat may pose health risks for hunters and associated consumers.

## Introduction

Lead (Pb) is a toxic non-essential metal that negatively affects multiple body systems in vertebrates, especially the nervous, cardiovascular and renal systems (Pokras and Kneeland, 2008). Children are particularly vulnerable to the effects of lead as they are considered to absorb a higher proportion of dietary lead than adults, and their developing nervous system is very sensitive to its effects (European Food Safety Authority, 2010; Naranjo et al., 2020). There is no level of exposure to lead known to be without harmful effects in humans (World Health Organization, 2022). In recent years, recognition of the health risks posed to certain human consumers by residues from lead-based ammunition in meat from wildlife species (game meat) has grown rapidly worldwide (Pain et al., 2022; Thomas et al., 2022). Dietary exposure to lead from ammunition also poses health risks to domestic animals such as hunter’s dogs (Fernández et al., 2021) and wildlife, including scavenging or predatory species (Pain et al., 2019) and waterfowl.

Lead gunshot and bullets frequently leave small lead fragments embedded in the meat of wild-shot game animals, many of which are too small and/or too numerous to be detected and removed during food preparation and mastication (Hunt et al., 2009; Kollander et al., 2017; Leontowich et al., 2022; Pain et al., 2010). Some ingested lead is absorbed from the intestine into the blood, and a causal relationship between consumption of lead-contaminated game meat and increase in blood lead levels (BLLs) has been shown experimentally in pigs (Hunt et al., 2006). There is a well-established association between increased BLLs in humans and consumption of game meat harvested with lead-based ammunition, in proportion to the amounts of game consumed (Berky et al., 2022; Meltzer et al., 2013; Tammone et al., 2021). Cooking processes using acid ingredients (wine, vinegar or marinades) can further solubilise lead particles (Mateo et al., 2011; Mateo et al., 2007; Schulz et al., 2021).

An additional risk, particularly for frequent consumers of game hunted with lead gunshot and subsistence hunting communities, is the potential for shot to be retained in the gastrointestinal system, intra-luminally and/or in the appendix (Tsuji and Nieboer, 1997). This can result in prolonged absorption of lead and consequent elevated blood lead concentrations (Madsen et al., 1988), and, very rarely, be associated with appendicitis (Larsen and Blanton, 2000). People frequently consuming wildlife harvested with lead ammunition and groups particularly vulnerable to the effects of lead (children and pregnant women) are at highest risk of associated negative health effects (Wani et al., 2015).

Studies from the past two decades have demonstrated high lead concentrations in a wide variety of game meat products. These have included game animals harvested with shotguns, including birds such as thick-billed murre (*Uria lomvia*) in Greenland (Johansen et al., 2001) and impala (*Aepyceros melampus*) in South Africa (Nkosi et al., 2022), white-tailed deer (*Odocoileus virginianus*) in the USA (Wilson et al., 2020), and a variety of other species of game bird and small mammals across Europe (Pain et al., 2010). Data from mammals harvested with rifle bullets includes ground venison from white-tailed deer donated to shelters in the USA (Cornatzer et al., 2009; Totoni et al., 2022), moose (*Alces alces*) from Norway (Lindboe et al., 2012), roe deer (*Capreolus capreolus*) from Germany (Schulz et al., 2021), chital (axis) deer (*Axis axis*) from Argentina (Tammone et al., 2021) and meat sauce made from wild pigs (*Sus scrofa*) sold commercially in Italy (Lenti et al., 2021). Studies have also been proposed on venison harvested by recreational hunters in New Zealand (Buenz et al., 2016). However, no published studies have assessed game meat products from Australia (Hampton et al., 2018).

Australia has a history of overlooking heavy metal contamination that can impact on human health, and this trend has been observed for lead (Berger et al., 2019) as well as mercury (Hg) (Schneider, 2021). Australia has a large and diverse commercial game meat industry as well as extensive consumption of non-commercial game meat by recreational and indigenous hunters (Hampton et al., 2018). Despite the popularity of hunting (Moloney et al., 2022) and game meat consumption in Australia (Gressier, 2016), no published studies have documented lead concentrations in game meat products, although reviews have highlighted the need for such studies (Hampton et al., 2018). This knowledge gap persists despite broad awareness among Australians of the risks of dietary lead exposure via other sources, e.g. eggs from ‘backyard’ chickens in urban areas (Yazdanparast et al., 2022). There is, however, growing awareness of the issue amongst the Australian recreational hunting community (Howlett, 2020). One recent study documented radiographic evidence of lead ammunition residues in a single game bird species (Hampton et al., 2022c), but did not quantify lead concentrations in the meat of these birds. Like most countries (Nkosi et al., 2021; Thomas et al., 2020), Australia has a maximum level of 0.1 mg/kg (ppm) wet weight (w.w.) for meat from cattle, sheep, pigs and poultry (excluding offal), but no level is set for game meat (Food Standards Australia New Zealand, 2017).

### Hypotheses and objectives

Here, we aimed to estimate the frequency and magnitude of lead contamination in Australian game meat. This study is the first to address this knowledge gap. We assessed lead contamination levels in four types of game meat using radiography and inductively coupled plasma mass spectrometry (ICP-MS). Our null hypothesis was that all game meat types had negligible lead contamination as measured via radiography or ICP-MS.

## Materials and methods

### Commercially available game meat products

The commercial harvest of macropods (kangaroos and wallabies) involves the shooting of millions of animals every year in Australia (1.4–1.7 million animals annually since 2010) (Australian Government, 2020). Commercially available kangaroo meat products derived from animals harvested from mainland Australia contain an unknown combination of the three legally harvested species: eastern grey (*M. giganteus*), western grey (*M. fuliginosus*) and red (*O. rufus*) kangaroos, all of which have adult animal mass typically 30–80 kg (Wynn et al., 2004). Bennett’s wallabies are confined to Tasmania in Australia (they also have an introduced range in New Zealand) and are much smaller than kangaroos, having an adult animal mass typically 10–25 kg.

To our knowledge, lead-based bullets are currently used for all macropod shooting (Hampton and Forsyth, 2016; Hampton et al., 2022d; Woodford et al., 2020). Typically, .223 calibre centrefire rifles are used to fire 55 grain ‘varmint’ style frangible bullets (Hampton and Forsyth, 2016). There are strict requirements for ‘head shooting’ of macropods (AgriFutures Australia, 2020), with a ‘zero tolerance’ policy applied by processors to body-shot animals (Wilson and Edwards, 2019). The majority of meat taken for human consumption from macropod carcasses is taken from the hind limbs (Wynn et al., 2004). In addition, at least some macropod processors screen all meat via X-ray with the aim of detecting metal contamination (Wilson and Edwards, 2019). However, this approach is unlikely to be fool proof, with recent research using synchrotron radiation having shown that many fragments from lead-based bullets are far too small to visualise via traditional X-rays (Leontowich et al., 2022).

Samples were taken from minced meat from kangaroos (*Macropus* and *Osphranter* spp.; *n*=36) and Bennett’s wallabies (*Notamacropus rufogriseus*; *n*=28) sold for human consumption at supermarkets (Table 1).

**Table 1:** Details of samples collected to represent four types of game meat from Australia for analysis of lead contamination

**Generic name**	**Wildlife species**	**State/s of Australia**	**Meat type**	**Sample size**	**Type of shooting**	**Source**
Kangaroo	Unknown mix of kangaroo species (*Macropus* and *Osphranter* spp.)	Unknown	Burgers	36	Commercial	Supermarket
Wallaby	Bennett’s wallaby (*Notamacropus rufogriseus*)	Tasmania	Mince	28	Commercial	Supermarket
Venison	Sambar (*Cervus unicolor*), fallow (*Dama dama*), and chital (*Axis axis*) deer	Victoria, South Australia	Mince, burgers, sausages	32	Recreational	Private hunters
Quail	Stubble quail (*Coturnix pectoralis*)	Victoria	Minced breast meat	37	Recreational	Private hunters

### Stubble quail from recreational hunters

Stubble quail (*Coturnix pectoralis*) are small (~100 g) ground-dwelling galliforms (Hampton et al., 2022c) and are one of very few native non-waterfowl bird species that can be legally hunted in Australia, and the only such species that can be hunted in the state of Victoria. They are commonly harvested using shotguns and gundogs on privately owned agricultural land and public lands (game reserves) (Ray et al., 2022). Currently, approximately 200,000 stubble quail are harvested annually in Victoria by approximately 28,000 licenced hunters (Moloney et al., 2022); all are destined for human consumption, and most are hunted with lead shot (Hampton et al., 2022c). While lead shot has been banned for waterfowl hunting in many (but not all) Australian jurisdictions, its use remains legal for non-waterbirds including grassland birds (Hampton et al., 2018) such as the stubble quail. A 2020 survey of Victorian game licence holders revealed that an estimated 81% (95% CI = 76–84%) of stubble quail hunters still use lead shot (Victorian Game Management Authority, 2020).

A total of 37 entire stubble quail harvested with lead shot were donated by two recreational hunters (Table 1). Details for these specimens are provided in Hampton et al. (2022c). We removed breast meat as per standard butchering methods, and then extracted obvious lead shot/pellets as these would usually be removed as part of butchering (plucking/skinning) or by consumers at the table. We then homogenised the whole breasts for each bird (minus the removed shot/pellets) in a food processor, removed a 5-g aliquot and sent this this off for analysis.

### Venison from recreational hunters

Australia has six species of wild introduced deer (Davis et al., 2016). Meat from three of these species were sampled in this study, sambar deer (*Cervus unicolor*), chital deer (*Axis axis*) and fallow deer (*Dama dama*). The sambar is Australia’s largest deer species (adults are typically ~180 kg) (Hampton et al., 2022b); chital and fallow deer are considerably smaller, with typical adult mass ~50 kg (Bengsen et al., 2021; Hampton et al., 2021a). Recreational deer hunting is a popular pastime in south-eastern Australia, with increasing trends observed in deer abundance (Watter et al., 2020) and total harvest by hunters (Moloney et al., 2022).

In each Australian state where recreational deer hunting is permitted, regulations stipulate minimum firearm and ammunition requirements. In Victoria, regulations specify a minimum calibre of .270 (6.85 mm) for centrefire rifles and a minimum projectile mass of 130 grains (8.45 g) (Chief Parliamentary Counsel, 2012). Deer in Australia may be shot with bullets made of any metal, including lead-based bullets, although some hunters choose to use lead-free (copper-based) bullets (Hampton et al., 2022b). Hunters typically aim to shoot deer in the thorax (‘chest shooting’), as for most large ungulate species globally (Stokke et al., 2018). The deer used to make the venison products we studied here were harvested using two different recreational hunting methods: stalking (sambar, fallow and chital deer) (Comte et al., 2022) and ‘hound hunting’ (sambar deer only) (Hampton et al., 2022a). We were not able to record the anatomical locations of deer from which the samples were taken before mincing.

Minced venison samples (*n*=32) were donated by three recreational hunters and comprised the three deer species described above (Table 1).

### Radiography

All meat packages were studied by digital radiography for qualitative detection of metal fragments, assumed to be lead fragments from bullets (Cornatzer et al., 2009). Minced meat was X-rayed for kangaroo, wallaby and venison, but whole quail breasts were X-rayed before mincing. The X-ray configuration involved a Toshiba Model TF-6TL-6 generator (Toshiba, Tokyo, Japan) with an Agfa digital plate and processor (Agfa-Gevaert Group, Mortsel, Belgium). The factors we used were 60 kV, 160 mA, 25 ms and a 100-cm film to focal distance. Digital Imaging and Communications in Medicine (DICOM) files were then analysed. We used Radiant and ImageJ software to visualise, enumerate and measure (maximum length in mm) metal fragments (Green et al., 2022; Leontowich et al., 2022; Wilson et al., 2020). At maximum magnification, the minimum fragment size that could be confidently detected in these DICOM files was 0.2 mm. Any sufficiently large fragments (>3 mm in length) were dissected from the meat, cleaned and had their elemental composition determined via portable X-ray fluorescence (XRF), as per the methods of Hampton et al. (2021b).

### ICP-MS measurements

Standard ICP-MS procedures were employed. Testing was performed at the School of Sciences Analytical Facility laboratory at Edith Cowan University, in Perth, Australia. These methods are described in Lohr et al. (2020) and Pay et al. (2021). Briefly, meat samples were dried to a constant weight and underwent acid digestion using a microwave digestion system. Lead concentrations were then determined using an iCAP Q ICP-MS (Thermo-Fisher Scientific, Omaha, USA) coupled to an ASX-520 AutoSampler. Certified Reference Materials were used as positive controls.

Calibration standards and continuing calibration verification solutions were prepared by diluting the stock solutions in ultrapure water. Quality controls included procedural blanks, continuous calibration verification and sample analysis duplicates (ca. 10%): every 10th sample was re-analysed for a duplicate read, with an average relative standard deviation (RSD) of 3.6%. ICP-MS results were reported as dry weight to account for different moisture levels in meat products that had spent different durations frozen. To compare our results to other published studies, we used a wet weight conversion factor of 0.1 μg/g w.w. ≈ 0.32 μg/g d.w. as per Mateo et al. (2014).

### Statistical analysis

Descriptive statistics were used to assess the frequency of lead pellets and fragments, and the distribution of ICP-MS data. Boxplots were prepared using the ggplot2 package (Wickham, 2016) in R version 4.2.1 (R Core Team, 2022), and two-by-two tables and Fisher’s exact test statistic were calculated using the epiR library (Stevenson et al., 2022) to assess the association between radiographically positive samples and samples with lead levels above the threshold of 0.1 mg/kg w.w. . We estimated diagnostic sensitivity and specificity for radiography considering ICP-MS as the reference (i.e. ‘gold standard’) measure of lead contamination. We did not attempt to statistically compare the influence of harvesting methods (commercial and recreational) or of animal species due to the confounding effects of interactions between these two variables in our dataset.

## Results

**Fig. 1:**
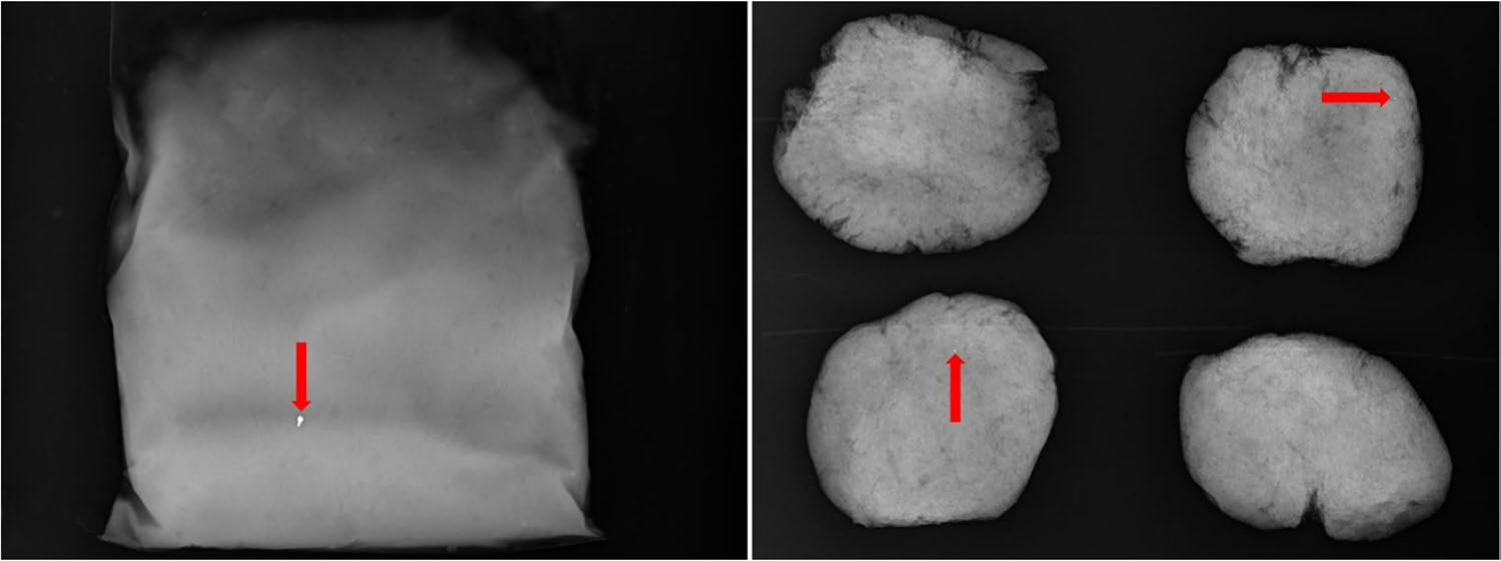
Radiographs of Australian game meat contaminated with metallic fragments (red arrows) assumed to be lead. The radiograph on the left shows a commercially available packet of wallaby mince containing a metallic fragment 3.7 mm long. The radiograph on the right shows four burgers made from deer meat (venison) by a recreational hunter, two of which contain metallic fragments 0.6–0.7 mm long

In total, we examined 133 minced game meat items of four types. Radiography revealed no visible fragments in any commercially available kangaroo products. One commercially available wallaby product contained a single, relatively large (3.7 mm maximum length; Fig. 1) fragment that was determined to be lead-based using portable XRF (>600,000 ppm lead). Radiographically visible pellets and/or fragments were detected in recreationally harvested meat types at frequencies of 35% and 28% for quail and venison (Fig. 1), respectively. Two out of 37 (5%) quail breasts contained whole shot pellets that were undetected during butchering but detected with X-ray. The mean number of fragments detected in positive items was 5.5 ± 4.5 for quail and 1.9 ± 1.9 for venison. Mean fragment size was 0.5 ± 0.4 mm for quail and 0.7 ± 0.3 mm for venison.

**Fig. 2:**
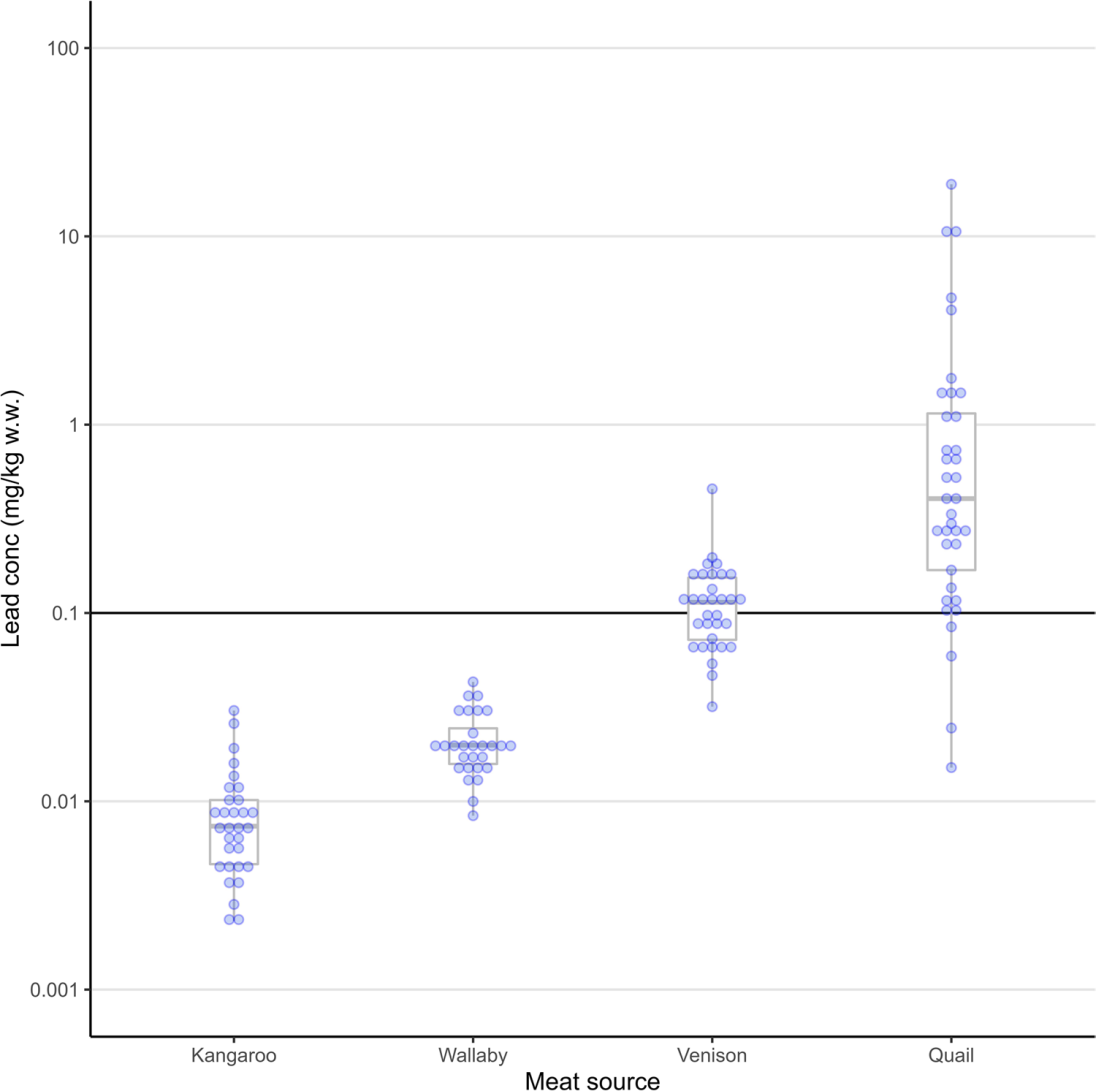
Boxplots on the log scale showing lead concentrations (mg/kg wet weight), as determined by inductively coupled plasma mass spectrometry (ICP-MS), in four types of game meat from Australia, from left to right: kangaroo, wallaby, venison and quail. The solid back horizontal line shows the Australia and New Zealand food standards threshold level for livestock meat of 0.1 mg/kg w.w.

Arithmetic mean lead concentrations in each meat type (mg/kg wet weight) were 0.01 ± 0.01 for kangaroo, 0.02 ± 0.01 for wallaby, 0.12 ± 0.07 for venison and 1.76 ± 3. 76 for quail (Table 2, Fig. 2). The frequency of samples exceeding the food standards threshold level of 0.1 mg/kg w.w. (Food Standards Australia New Zealand, 2017) were zero for kangaroo and wallaby, but 53% (95% CI: 35%, 71%) for venison and 86% (95% CI: 71%, 95%) for quail (Table 2).

**Table 2:** The frequency of metallic fragments seen in radiographs and lead concentrations (mg/kg wet weight), as determined by inductively coupled plasma mass spectrometry (ICP-MS), in four types of game meat from Australia (for details of each meat type, see Table 1)

**Generic name**	**Radiography**		**Lead concentrations via ICP-MS (mg/kg wet weight)**					
	**Frequency of samples with metallic shot and/or fragments (%)**	**Mean ± SD number of fragments for positive samples**	**Mean**	**SD**	**Min**	**Max**	**Frequency of samples >0.1 mg/kg (%)**	**Maximum factor of 0.1 mg/kg**
Kangaroo	0	NA	0.01	0.01	<LOQ	0.03	0	0.30
Wallaby	4	1.0 ± 0.0 (*n*=1)	0.02	0.01	0.01	0.04	0	0.43
Venison	28	5.5 ± 4.5	0.12	0.07	0.03	0.46	53	4.56
Quail	35	1.9 ± 1.9	1.76	3.76	0.02	18.90	86	188.98

Of 23 radiographically positive samples, 17 had lead levels above the threshold of 0.1 mg/kg w.w., as did a further 32 samples that were radiographically negative. Seventy-eight further samples were radiologically negative and below the threshold. Radiography therefore had a diagnostic sensitivity of 35% (95% CI: 22%, 50%) for detecting samples that were above the food standards threshold and 93% diagnostic specificity (95% CI: 85%, 97%).

For individual meat types, there was no significant relationship between radiographically positive samples and those that exceeded the threshold concentration of lead in meat, for venison (Fisher exact test; *P*=0.6989) or quail (Fisher exact test; *P*=0.1398). This relationship could not be tested for kangaroo or wallaby meat due to the absence of samples exceeding the threshold concentration of lead in meat.

## Discussion

To our knowledge, this is the first study to report lead contamination levels in Australian game meat products. The lead concentrations increased by approximately an order of magnitude from macropods (kangaroos and wallabies: mean 0.01–0.02 mg/kg) to venison (mean 0.12 mg/kg) to quail (mean 1.76 mg/kg). Lead concentrations in those samples analysed suggest little risk of harmful lead exposure for human consumers of commercially available game meat products in Australia, although lead fragments were still present in wallaby meat and our sample size was relatively small. However, Australian hunters and their families are being exposed to elevated amounts of lead through consuming hunted meat, as occurs worldwide. This risk is higher for consumers of stubble quail, the only avian species and the only species in our study harvested with shotguns.

The finding that radiography had a diagnostic sensitivity of only 35% for detecting samples that were above the food standards threshold (0.1 mg/kg w.w.) is biologically important. Put another way, radiography failed to detect more than half of the samples with lead concentrations exceeding the threshold of concern at the resolution used in our study (minimum detectable fragment size of 0.2 mm). This is probably due to the presence of small lead particles and also tiny ‘nanoparticles’ that are too small to detect on a radiograph (Kollander et al., 2017; Leontowich et al., 2022). This result confirms that traditional radiography (at the resolution used in our study) is not necessarily a reliable method for screening game meat products for lead contamination. In other words, the absence of visibly detectable metallic fragments in X-rays does not guarantee that game meat products contain non-harmful lead concentrations. Despite these limitations, radiography has been relied on widely to screen game meat samples for lead contamination (Cornatzer et al., 2009) and has been proposed as an adequate protective measure for lead contamination in kangaroo meat products (E. Buckle, personal communication). This reliance warrants re-consideration.

Mean lead concentrations in stubble quail (1.76 mg/kg w.w.) were an order of magnitude higher than the next-highest game meat type (venison). The vast majority of samples (86%) also exceed the food standards maximum levels of contaminants threshold level of 0.1 mg/kg w.w. (Food Standards Australia New Zealand, 2017). These results fall within the range of values reported from gamebirds and small mammals killed with gunshot in Europe (Pain et al., 2022). As all harvested and retrieved stubble quail are consumed by humans, the continued use of lead gunshot may present a health risk to consumers, especially frequent consumers and groups particularly vulnerable to the effects of lead, especially children (Hampton et al., 2022c). In spite of these risks and the long-term popularity of hunting stubble quail in south-eastern Australia (Kinghorn, 1926), no data relating to lead contamination have previously been published for this species. However, a recent paper described the radiographic distribution of embedded shot and fragments in stubble quail (Hampton et al., 2022c), and called for data on lead concentrations in the edible meat of the species to complement these findings.

Although the mean contraction of lead in venison was an order of magnitude lower than in meat from stubble quail, over half of the samples we examined contained lead concentrations that exceeded the legal limit for meat in Australia (Food Standards Australia New Zealand, 2017). Despite this concerning finding, the mean concentrations found in minced venison in Australia were actually relatively low compared with those from similarly harvested and prepared minced cervid meat in some studies from other countries. *Minced* (as opposed to non-minced) cervid meat has been shown to be associated with a significant increase in the risk of harmful human lead exposure (Meltzer et al., 2013). Notably, a mean lead concentration of 5.6 ppm w.w. (~50× higher than our data) was found in 52 minced meat packages donated by recreational moose (*Alces alces*) hunters in Norway (Lindboe et al., 2012). A mean lead concentration of 1.8 ppm w.w. (~15× higher than our data) was found in 98 minced meat packages donated by recreational white-tailed deer hunters in the USA (US Department of Health and Human Services, 2008).

From non-minced cervid meat, lead contamination is much more variable. For example, lead concentrations found in haunch (rump/hindlimb) meat from 745 roe deer and 64 red deer (*Cervus elaphus*) in Germany were 0.169 ppm w.w. (Gerofke et al., 2018) and 0.0151 ppm w.w. (Martin et al., 2019), respectively. Meanwhile, meat taken from near the wound channel in the same two studies had concentrations of 13.958 and 15.82 ppm w.w., respectively. Similar results have been reported for chital (axis) deer from Argentina (Tammone et al., 2021) and red deer from Poland (Dobrowolska and Melosik, 2008). Much of this variation can be explained by the heterogeneous distribution of lead fragments within any cervid shot (Tsuji et al., 2009; Wilson et al., 2020). The amount of tissue contamination is dependent on the distance between the muscle sampling site and the anatomical location of where the animal was shot, as well as whether the bullet struck bone (Broadway et al., 2020; Müller-Graf et al., 2017; Schlichting et al., 2017). In contrast, it is likely that mincing homogenises any lead contamination within meat (Thomas et al., 2020) and is reflective of what most venison consumers actually eat.

These results from venison samples have important ramifications for a considerable number of Australians. In Victoria alone, ~50,000 people were licenced to hunt deer in 2021, harvesting ~119,000 deer (Moloney and Flesch, 2022). In addition, commercial venison harvesting has recently been legalised in some Australian states (Watter et al., 2020), coinciding with anecdotal evidence of a recent increase that has been reported in the popularity of hunted wild game meat among consumers in many global regions (Marescotti et al., 2019), including Australia (Gressier, 2016). Unfortunately, we did not examine any commercially harvested Australian venison products in this study. Nonetheless, the numbers of people likely exposed to venison with lead concentrations exceeding legal limits for other types of meat in Australia are substantial.

The finding of negligible lead contamination in commercially available macropod meat will be reassuring to consumers and conservationists promoting the ecological benefits of commercial macropod harvesting (Read et al., 2021). It is likely that the strict use of head shots for kangaroo and wallaby species is highly effective at preventing lead fragments from dispersing into the valuable meat-producing muscle of the hindquarters (Wynn et al., 2004). The X-ray screening of macropod meat products by at least some processors (Wilson and Edwards, 2019) is also likely to reduce the prevalence of contaminated products being sold, although we found that radiography only detected 35% of samples that were above the food safety threshold. Macropod meat (especially kangaroo) is also used extensively for pet (mostly dog) food in Australia (Spiegel and Greenwood, 2019), where food safety and hygiene regulations are less stringent (Salmon et al., 2022). It is unknown what levels of lead contamination are typically found in macropod-based pet meat in Australia. Given the risks to dogs fed trimmings from game killed with lead-based bullets (Fernández et al., 2021; Høgåsen et al., 2016), this should be a topic for future research.

There were important limitations to our study. First, we chose only four game meat types to analyse for logistical and budgetary reasons, whereas Hampton et al. (2018) described at least 16 Australian wildlife species harvested with lead-based ammunition from which game meat is commonly consumed. Notable species that we did not assess include feral pigs/wild boar (Lambré et al., 2022) and European rabbits (*Oryctolagus cuniculus*) (Hampton et al., 2020). Second, our samples sizes were relatively small (*n*<50 for each meat type). Third, we did not determine the elemental composition of metallic fragments in radiographs, but rather assumed them to be lead. Fourth, we assessed different types of game meat products. Some products contained ingredients other than game meat (e.g. venison sausages), and some did not (i.e. quail breast). Some meat products had been frozen for short versus long durations, which likely affected their moisture levels (Lee et al., 2008). Nonetheless, the results show unequivocally the risk of harmful lead exposure associated with the frequent consumption of some Australian game meat types. This issue requires further attention and the limitations of our study provide opportunities for further work.

In conclusion, our results illustrate that some Australian commercial game meat products contain low lead concentrations that are not currently considered of health concern. In contrast, some recreationally harvested game meat contains high average lead concentrations that pose health risks for frequent consumers and vulnerable groups, e.g. hunters, their families and associates, and children. From a One Health perspective (Arnemo et al., 2022), these health impacts should be considered in addition to those imposed on wildlife scavengers that ingest lead from the discarded carcasses of the animals harvested for human consumption (Hampton et al., 2022d). The issue of harmful lead exposure in Australian consumers of wildlife meat deserves further scientific scrutiny.
